# Can water, sanitation and hygiene help eliminate stunting? Current evidence and policy implications

**DOI:** 10.1111/mcn.12258

**Published:** 2016-05-17

**Authors:** Oliver Cumming, Sandy Cairncross

**Affiliations:** ^1^ Department of Disease Control, Faculty of Infectious and Tropical Diseases London School of Hygiene and Tropical Medicine London UK

**Keywords:** sanitation, water, stunting, child nutrition, child public health, early growth

## Abstract

Stunting is a complex and enduring challenge with far‐reaching consequences for those affected and society as a whole. To accelerate progress in eliminating stunting, broader efforts are needed that reach beyond the nutrition sector to tackle the underlying determinants of undernutrition. There is growing interest in how water, sanitation and hygiene (WASH) interventions might support strategies to reduce stunting in high‐burden settings, such as South Asia and sub‐Saharan Africa. This review article considers two broad questions: (1) can WASH interventions make a significant contribution to reducing the global prevalence of childhood stunting, and (2) how can WASH interventions be delivered to optimize their effect on stunting and accelerate progress? The evidence reviewed suggests that poor WASH conditions have a significant detrimental effect on child growth and development resulting from sustained exposure to enteric pathogens but also due to wider social and economic mechanisms. Realizing the potential of WASH to reduce stunting requires a redoubling of efforts to achieve universal access to these services as envisaged under the Sustainable Development Goals. It may also require new or modified WASH strategies that go beyond the scope of traditional interventions to specifically address exposure pathways in the first 2 years of life when the process of stunting is concentrated.

## Introduction

This article was inspired by the ‘Stop Stunting’ Conference held in Delhi last year to convene actors from multiple countries and sectors to address a shared concern: the enduring and seemingly intractable challenge of childhood stunting in South Asia. Huge progress has been made in much of the South Asia region in extending healthcare, education and economic opportunity, and these investments have brought dramatic improvements in maternal and child mortality, in school retention rates and in overall economic output. Despite this laudable progress, the prevalence of childhood stunting in South Asia remains high with profound consequences for those children affected: increasing their susceptibility to infectious disease morbidity and mortality, diminishing their future educational achievements and reducing their economic productivity in later life. The failure to address stunting in South Asia, and other high‐burden regions, stands to undermine progress in other sectors and trapping future generations in poverty and ill health.

Stunting is a complex problem as depicted by various conceptual frameworks, focused on ‘child malnutrition’ (UNICEF 1990), ‘maternal and child undernutrition’ (Black *et al.* 2013) and ‘food and nutrition security’ (Gross *et al.* 2000). The causes of stunting are multifactorial and inter‐linked, spanning biological, social and environmental spheres. Water, sanitation and hygiene (WASH), the focus of this paper, feature at various levels in these frameworks with varying degrees of proximity to the outcome of stunting, as immediate or proximate risk factors but also as more distant causes or determinants of stunting. For example, different aspects of WASH have been plausibly linked to all four ‘pillars’ of the food and nutrition security framework (Cumming *et al.* in press): food ‘availability’, through water as a resource for agricultural production; food ‘access’, through household income diverted from food by the cost of obtaining water and ensuring adequate sanitation; food ‘stability’, through the economic shock of treating related infectious disease or associated inability to work; and lastly food ‘utilization’, through the effect of WASH‐related enteric infections on the body's ability to utilize the available nutrients.

Two broad questions emerge for those considering WASH as a potential component of more effective comprehensive strategies to address stunting. Firstly, can WASH interventions make a significant contribution to reducing the global prevalence of childhood stunting? Secondly, and if so, how can WASH interventions be delivered to optimize their effect on stunting and accelerate progress? These questions are of importance to both the WASH and nutrition sectors, and for wider debates concerning the allocation of scarce resources available for improving public health and other social outcomes in low and middle‐income countries where the burden of stunting is highest.

Here, we review how poor water, sanitation and hygiene can influence the process of stunting through biological and social mechanisms and then consider the strength of evidence available for an effect of these interventions on stunting. Secondly, we identify the underlying parameters that might plausibly govern the degree to which WASH interventions reduce the risk of stunting and then discuss the implications for practitioners and policymakers concerned with mobilizing WASH resources in support of broader efforts to reduce stunting.

### Water, sanitation and hygiene

The importance of safe drinking water, sanitation and hygiene (WASH) has long been recognized with regard to public health in general and the health of infants and young children in particular (Jones 1923). Indeed, the birth of ‘public health’ as a defined area of public policy and as a professional discipline is now synonymous with these endeavours to improve ‘sanitary conditions’, following the pioneering work of Chadwick (1842), Farr (1866) and Snow (1855) in the 19th century. WASH is often divided into four rather than three categories, with ‘water’ interventions divided into two subcategories: ‘water quantity’ and ‘water quality’. The former describes interventions that improve the quantity of drinking water available to the household, and the latter describes interventions that improve the microbial quality of drinking water, whether this is at the water source or at the point of use or consumption. Sanitation concerns technologies and behaviours that serve to safely contain excreta, preventing human contact, and hygiene is commonly used to mean washing with soap at critical times (e.g. after defecation and before eating).

**Figure 1:**
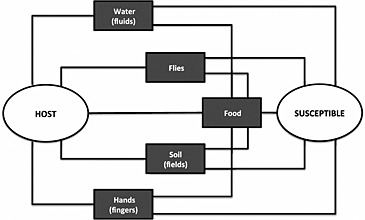
The ‘F‐diagram’. Source: Adapted from Wagner & Lanoix 1958 and Kawata 1978.

These public health interventions together form an interlocking set of barriers that prevent exposure to disease‐causing organisms via five transmission pathways as famously depicted in the ‘F‐diagram’ (Fig. 1) of Wagner & Lanoix (1958). The interdependency of these barriers is well illustrated by the cholera outbreak investigated by John Snow in Soho, London, almost two centuries ago (Snow 1855). The index case was an infant whose infected stools were emptied into a poorly constructed cesspool, which contaminated the water source that served the now infamous Broad Street water pump (Johnson 2008). While Snow's work elegantly demonstrated that cholera was transmitted from host to susceptible individual by the medium of water, the epidemic itself had as much to do with the prevailing sanitation infrastructure and hygiene behaviours as it did with the water supply.

Although WASH interventions are often described in terms of their role in preventing disease transmission, the benefits are not confined to health. Improvements in water supply often serve to reduce the distance travelled to the water source leading to significant time savings for poor households that can transform the lives of the women and children whose responsibility it largely is to collect water [World Health Organization (WHO) & UNICEF 2010]. A senior World Bank economist famously argued that these benefits alone provide sufficient economic justification for the investment costs of water supply without any consideration of the health benefits that may accrue (Churchill *et al.* 1987). The non‐health benefits of sanitation include privacy and convenience afforded by improved facilities. There is now a growing literature that documents that this lack of ‘privacy and convenience’ can lead to an increased risk of violence, whether this is physical, sexual and psychological, that is borne primarily by women. It is perhaps because of these risks that shared and public sanitation facilities have been found to be less preferable to women as compared with men (Biran *et al.* 2011).

### Global coverage for water, sanitation and hygiene

The WHO/UNICEF Joint Monitoring Programme tracks progress against target 7.c of the Millennium Development Goals (MDG): ‘to reduce by half the proportion of the population without sustainable access to safe drinking water and improved sanitation by 2015’. At a global level, it has been announced that while the water component of this MDG target was met in 2010, the sanitation target has been missed by a substantial margin.

In most countries defined as low and middle income (LMIC) (Group 2015), most people lack household‐level access to a safe and reliable supply of drinking water, and to a safe and acceptable form of sanitation (WHO & UNICEF 2014). Globally, it has been estimated that over one‐third of the world's population are without these services at home (Cumming *et al.* 2014). While challenges persist in other regions, sub‐Saharan Africa and South Asia account for the greatest deficits in access to safe water and sanitation (WHO & UNICEF 2014). Access to, or more appropriately the practice of, safe hygiene is much harder to estimate and is not currently reported at a global level. The most comprehensive published analysis to date, based on the results of a systematic review of studies reporting observed handwashing practice, estimated that fewer than one in five people globally wash their hands with soap after defecation (Freeman *et al.* 2014b).

Analysis of historical progress and current coverage reveals marked geographic and social disparities in access to these services. Between countries (WHO & UNICEF 2014) but also within many countries (Pullan *et al.* 2014), access to safe water and sanitation varies significantly. Disparities in access between rural and urban communities are well documented, with access to both water and sanitation services in rural generally much lower than in urban areas, especially in LMIC (Bain *et al.* 2014b). Viewed at the level of mean global averages, the differences between urban and rural areas are striking: in 2012, there were 500 million more people without access to safe water in rural areas vs. urban areas, and 1 billion more without access to sanitation (WHO & UNICEF 2014). However, disparities in access between the poorest quintiles for rural and urban populations are far less marked (Rheingans *et al.* 2013).

More than half of the world's population now reside in urban areas, and over one‐third of these urban dwellers live in ‘slums or informal settlements’ with that proportion being much higher in LMIC Development, W. H. O. C. F. H., & Programme, U. N. H. S. (2010). Although access to safe water and sanitation is generally higher in urban vs. rural areas (Bain *et al.* 2014b), the proportion of the urban population with access to safe services is actually falling as investment fails to keep pace with urban population growth (WHO & UNICEF 2015). It has long been recognized that the risk of enteric infection may be greatest in poor urban areas due to the combination of high population density and limited infrastructure (White *et al.* 1972), which is supported by studies looking at certain soil‐transmitted helminth infections (Strunz *et al.* 2014b) and diarrhoea (Mock *et al.* 1993) and childhood undernutrition (Olack *et al.* 2011). A failure to target investments at the growing population living in informal areas may undermine progress on reducing child mortality in some countries (Fotso *et al.* 2007; Rheingans *et al.* 2013).

The global prevalence of childhood stunting has declined considerably during the MDG period: while in 1990 40% of children globally were estimated to be stunted (height for age z‐score [HAZ]< −2), it is now estimated that this has fallen to below a quarter (Black *et al.* 2013). In absolute terms, the number of children with stunting has fallen by approximately 100 million, although this still leaves 150 million children stunted today (Black *et al.* 2013). As with the shortfall in water and sanitation coverage, the global burden of stunting is heavily concentrated in just two regions of the world: South Asia and sub‐Saharan Africa.

### The broader infectious disease burden attributable to WASH

Safe WASH is of paramount public health importance *without* considering the plausible impact on childhood stunting. Improved access to WASH can prevent a large infectious disease burden that includes diarrhoeal diseases but also other important infectious diseases. Diarrhoeal disease, encompassing a broad range of bacterial, viral and protozoal enteric infections, and largely preventable with improved WASH, was ranked as the fourth leading cause of disability globally in 2010, after ischaemic heart disease, lower respiratory heart infections and strokes (Murray *et al.* 2013).

A recent series of papers by a WHO‐led group of experts quantified the global diarrhoeal disease burden attributable to poor water, sanitation and hygiene (Bain *et al.* 2014a; Freeman *et al.* 2014a; Prüss‐Ustün *et al.* 2014; Wolf *et al.* 2014). The authors estimated that approximately 500 000, 280 000 and 300 000 deaths are attributable to poor water, sanitation and hygiene, respectively (Prüss‐Ustün *et al.* 2014). Using a formula for the aggregate burden for a cluster of risk factors (Lim *et al.* 2013), the total diarrhoeal burden of disease for WASH was estimated at over 800 000 deaths, equivalent to 1.5% of the total global burden of disease (Prüss‐Ustün *et al.* 2014). Almost half of these deaths were among children, with WASH accounting for 5.5% of the total burden of disease for this age group (Prüss‐Ustün *et al.* 2014), and diarrhoea remains a leading cause of child deaths globally and especially in high‐burden regions, such as sub‐Saharan Africa and South Asia (Liu *et al.* 2012).

Supported by evidence of variable quality, WASH is linked to a wide range of other infectious disease health outcomes, including helminth infections (Ziegelbauer *et al.* 2012; Strunz *et al.* 2014a), schistosomiasis (Grimes *et al.* 2014), trachoma (Stocks *et al.* 2014), respiratory infections (Rabie & Curtis 2006) and maternal and reproductive infections (Benova *et al.* 2014). Aggregating the disease burden for WASH – itself a cluster of overlapping risk factors – to take account of multiple and related outcomes (e.g. diarrhoea and pneumonia) is methodologically challenging. However, one recent WHO analysis that did this reported that approximately 10% of the total global burden of disease could be prevented with improved WASH (WHO 2008).

### Can safe water, sanitation and hygiene prevent stunting?

The pathways linking poor WASH to childhood stunting are complex, spanning multiple direct biological routes and many broader, less direct routes. To understand these, it is necessary to place the generally better investigated direct biological linkages within a broader socio‐economic framework which considers aspects such as accessibility and affordability of water supplies and sanitation facilities. Here, we first consider the biological mechanisms that plausibly link WASH and stunting, and then secondly, we consider the social and economic mechanisms.

#### Biological mechanisms

Three biological mechanisms, in particular, have been described that link poor WASH to undernutrition directly: (1) via repeated bouts of diarrhoea (Briend 1990; Checkley *et al.* 2008; Petri *et al.* 2008; Richard *et al.* 2013); (2) soil‐transmitted helminth infections, *Ascaris lumbricoides*, *Trichuris trichiura*, *Ancylostoma duodenale*, and *Necator americanus* (O'lorcain & Holland 2000; Prüss‐Üstün & Corvalán 2006; Hall *et al.* 2008; Ziegelbauer *et al.* 2012); and (3), a subclinical condition of the gut, referred to variously as tropical enteropathy (Baker & Mathan 1972; Humphrey 2009a), environmental enteropathy (Fagundes‐Neto *et al.* 1984; Korpe & Petri 2012) or, most recently, and as used here, environmental enteric dysfunction (EED) (Haghighi *et al.* 1997; Humphrey 2009b; Keusch *et al.* 2014; Crane *et al.* 2015). For each of these, the effect of WASH on undernutrition is mediated by exposure to enteric pathogens and symptomatic or asymptomatic infection.

Frequency of diarrhoeal disease, as a syndrome, irrespective of its causes, is strongly correlated with growth faltering (Checkley *et al.* 2003; Checkley *et al.* 2008). Demonstrating a causal relationship between diarrhoea and malnutrition though is challenging, as undernutrition can increase both the likelihood and severity of diarrhoea disease (Brown 2003; Caulfield *et al.* 2004). However, a recent pooled analysis of data from nine countries with longitudinal morbidity and anthropometry provides evidence that repeated bouts of diarrhoea cumulatively increase the risk of stunting in children (Checkley *et al.* 2008). These findings are consistent with the findings of various other studies (Esrey *et al.* 1985; Esrey *et al.* 1991; Prüss‐Üstün & Corvalán 2006; Guerrant *et al.* 2008). While the evidence is more limited, Petri identifies a number of studies linking specific diarrhoeagenic pathogens to malnutrition, including pathogenic *Escherichia coli*, *Shigella*, *Giardia* and *Cryptosporidium* (Petri *et al.* 2008)*.*

Soil‐transmitted helminth infections, or helminthiasis, can be prevented with adequate sanitation (Strunz *et al.* 2014b) and are strongly associated with childhood undernutrition (Prüss‐Üstün & Corvalán 2006). In particular, more severe cases of ascariasis and trichuriasis are associated with growth faltering in children (O'lorcain & Holland 2000; Hotez *et al.* 2004; Bethony *et al.* 2006). Hookworm infections during pregnancy can lead to malabsorption of nutrients and maternal anaemia, which in run are associated with stunting at birth (Black *et al.* 2013). Brooker and colleagues estimate that in sub‐Saharan Africa, over a quarter of all pregnant women are infected with hookworm (Brooker *et al.* 2008).

There is growing evidence linking symptomatic and asymptomatic enteric infections to EED. This syndrome was first described in the 1960s (Cook *et al.* 1969) and referred to as ‘Tropical Enteropathy’ (or ‘jejunitis’). The renaming to environmental enteropathy in the 1980s and 1990s (Fagundes‐Neto *et al.* 1984), and more recently to EED (Keusch *et al.* 2013; Keusch *et al.* 2014), reflects a growing appreciation of the role of the environment in the development of this condition. EED is an asymptomatic syndrome causing chronic inflammation, reduced nutrient absorption of the intestine and a weakened barrier function of the small intestine (Keusch *et al.* 2014; Crane *et al.* 2015). These abnormalities in gut function and structure may have profound consequences for affected children, including deficits in growth, early childhood development and immune function (McKay *et al.* 2010; Korpe & Petri 2012; Keusch *et al.* 2014; Crane *et al.* 2015). Although more research is needed, it has been argued that EED, and not diarrhoea, may be the primary causal mechanism linking WASH to child growth (Humphrey 2009a). One observational study in Bangladesh has shown that children living in households with improved WASH are both less likely to have EED, measured by lactulose : mannitol ratios in their urine [a measure of gut permeability (Lunn *et al.* 1991)], and are less likely to be stunted (Lin *et al.* 2013).

#### Social and economic mechanisms

Another important relationship is the energy cost of carrying water for long distances from the source to the home. White *et al.* (1972) estimated from various sources that the average woman, carrying a typical load of 20 L on level ground, would consume some 39 cal per kilogramme of body weight per hour. With an assumption that 1 g of maize meal yields 3.5 cal, the average cost of water in East Africa, where most people required less than an hour to collect water, was estimated as US$25 per year.

When the water‐carrying is performed by professional vendors, as is more often the case in urban areas, it is far more expensive to the consuming household. Typically, vendor prices are 10 to 20 times greater than the prices charged by the official water utility, amounting on average to some 20% of the household's income (Zaroff & Okun 1984). The prices may seem exorbitant, but this reflects the inefficiency of water transportation by such technologies as hand trolleys, donkey carts, jerry cans and buckets. If the vendors' prices are understandable in terms of their technology, how are we to understand the willingness to pay of the customers? Seen as a purchase of time, rather than water, the transaction is not as unfavourable as it might seem. Whittington *et al.* (1990) studied the options open to the customers of vendors in Ukunda, Kenya, and found that they usually chose the more costly, time‐saving option only if the trade‐off valued their time at more than the unskilled wage rate.

However, that does not per se render it economic for a poor family to opt for the water vendor over collecting water themselves because there may little or no spare income within the household budget to pay for water. The poorer the family, the less remains after food expenditure and so greater is the proportion of household expenditure devoted to food. This relationship is known as Engel's Law – not after Friedrich Engels, the co‐founder of Marxist theory, but for the 19th century Saxon Government accountant Ernst Engel (1821–1896) who first observed this relationship between income and food expenditure (Houthakker 1957).

**Figure 2:**
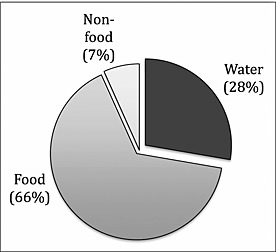
Typical breakdown of weekly household expenditure in low‐income areas of Khartoum, Sudan (1987). Source: Cairncross & Kinnear 1992.

The pie chart (Fig. 2) shows the breakdown of a typical weekly budget of a household in the low‐income areas around Khartoum, Sudan. It is striking that water already accounts for almost 30% of the household budget, and food two‐thirds of the budget. So, imagine for a moment that this is *your* family budget, and that the water price has just doubled; it is hard to see how to meet this need for additional but essential expenditure, without taking from the food budget.

Thus, water supply affects nutritional status not only via the complex metabolic links described in the previous text and elsewhere in this series of papers, but also by the most direct route imaginable: the high cost paid for water by the poorest – and the poor pay for water at by far the highest cost – which leaves them without insufficient funds for an adequate diet. Indeed, bearing in mind the impact of nutrition on mortality, many of the poor pay for water with their very lives.

The fact that poor WASH brings a risk of death from diarrhoeal disease may help to explain why people are willing to pay such a high price for water. Table 1 illustrates the inelasticity of demand for it. While the residents of Karton Kassala had to pay three times more than the people in Meiyo for their water, they used roughly the same amount of water per capita – if anything, slightly more. This lack of elasticity with regard to price was accompanied by income inelasticity of demand; households with a wide range of incomes were using roughly the same amounts of water.

**Table 1:** Inelastic demand; water prices and observed daily per capita water consumption in two low‐income areas of Khartoum, Sudan, 1987

	Meiyo (*n* = 22)	Karton Kassala (*n* = 28)
Mean† household size	7.3	8.3
Mean‡ income/head (Sudanese pounds/month)	42	47
Mean† water price (Sudanese pounds/drum)	1.50	4.64
Mean‡ water consumption (litres per capita per day)	24.2	27.0
Mean† % of income spent on water	16.5	55.6

Source: Cairncross & Kinnear 1992.

† Averaged by household, and

‡ averaged by individual.

These two findings have important policy implications. First, the income inelasticity means that the poorest households are paying the greatest proportion of their income for water, although they can least afford it. Second, the demand inelasticity means that price is highly sensitive to supply. Indeed, cases were found in Sudan where a slight constraint on the availability of water to fill the vendors' donkey carts led to a doubling or tripling of the price. The contrary is also true; facilitating the business of the water vendor (for example, by drilling more boreholes and offering credit to buy carts and donkeys) should lead to a substantial drop in the price of water. This drop in water prices will free up expenditure for the food budget, especially in the poorest households who most need it.

From this perspective, WASH can appear as a Holy Grail of community‐based nutrition projects: delivering savings for more food, particularly to the poorest. As water is regarded as ‘women's business’, the savings go directly into the pocket of the housewife and mother, the member of the household who may be best placed to ensure that children benefit. The lack of studies documenting this in the literature is evidence of the difficulty of cross‐sectoral vision and collaboration. Hopefully, we are now in more enlightened times, when nutritional benefits are achieved by interventions more subtle than handing out food.

### Experimental evidence for the effect of WASH interventions on stunting

Although a number of studies have found a significant association between access to improved WASH and improved growth after adjusting for confounding using a range of statistical methods (Esrey *et al.* 1985; Esrey *et al.* 1991; Spears 2013; Spears *et al.* 2013), a recent Cochrane review identified only five experimental intervention studies for the effect of WASH on undernutrition. These studies spanned different WASH interventions on childhood stunting: treatment of household drinking water by solar disinfection (Du Preez *et al.* 2010; Du Preez *et al.* 2011; McGuigan *et al.* 2011), chlorination (Luby *et al.* 2006), flocculants (Luby *et al.* 2006) and the provision of soap and promotion of handwashing (Luby *et al.* 2004). Critically, though, no water supply or sanitation interventions were identified. While pooled analysis found no effect of these WASH interventions on weight‐for‐age z scores and weight‐for‐height z‐scores, a small statistically significant effect was reported on height‐for‐age z scores [0.08 *z*‐score; 95% confidence interval: 0.00, 0.16] among participants under 5 years, with a larger effect for children under 2 years of age (0.25 *z*‐score; 95% confidence interval: 0.14, 0.35) in subgroup analysis.

Although no sanitation interventions were identified in this Cochrane review, five trials have subsequently published results describing the effect of sanitation interventions on stunting. Two of these studies (Hammer & Spears 2013; Pickering *et al.* 2015) reported significant effects on stunting, and three found no effect (Cameron *et al.* 2013; Clasen *et al.* 2014a; Patil *et al.* 2014). Notably, the interventions for those trials reporting no effect, two in India (Clasen *et al.* 2014a; Patil *et al.* 2014) and one in Indonesia (Cameron *et al.* 2013), had very low levels of uptake and compliance, which may explain their findings of no effect. By contrast, Pickering *et al.* report that access to sanitation increased substantially and open defecation reduced as a result of the intervention evaluated in Mali, West Africa (2015), while the intervention evaluated by Hammer & Spears in India achieved more modest increases in sanitation access (2013). This epidemiological literature confirms what is well known by many WASH implementers that the requisite changes in behaviour are hard to initiate and even harder to sustain over time.

At least three large WASH intervention studies are currently underway that will add to this evidence base and answer important outstanding questions (Humphrey 2013; Arnold *et al.* 2013; Brown *et al.* 2015). The factorial design of the Sanitation, Hygiene, Infant Nutrition Efficacy [SHINE] (Humphrey 2013) and WASH Benefits (Arnold *et al.* 2013) trials will permit the quantification of both the independent effect of WASH interventions on stunting and the combined effect of WASH and food supplementation interventions together. All three trials include biological markers of EED to assess whether improvements in WASH can reduce EED and to what extent the effects of WASH on stunting are mediated by this subclinical condition. Lastly, the interventions assessed in these trials have novel aspects, including the SHINE trial, which specifically addresses maternal and child environmental exposures, and the MapSan trial (Brown *et al.* 2015), which, for the first time, evaluates an urban on‐site sanitation intervention in high‐density informal settlements.

### How much stunting might be prevented with improved WASH?

The recent Lancet Series on child and maternal undernutrition came to the somewhat sobering conclusion that if it were possible to scale‐up 10 ‘evidence‐based nutrition interventions’ to almost complete coverage in the 34 countries that have 90% of stunted children, the global prevalence of stunting would be reduced by just one‐fifth (Bhutta *et al.* 2013). These findings along with those of other studies (Dewey & Aduafarwuah 2008) suggest that stunting is unlikely to be eliminated without addressing the underlying determinants of undernutrition alongside deficiencies in the quantity and quality of infant and child nutritional intake. This broad category of interventions that tackle the underlying determinants is sometimes referred to as ‘nutrition‐sensitive’ interventions and includes WASH but also things such as family planning services, maternal education and social safety nets (Black *et al.* 2013). As discussed in the previous text, WASH potentially impacts stunting through multiple and interacting biological and socio‐economic mechanisms that are difficult to assess independently.

At the level of public policy, internationally and nationally, much of the interest in WASH and undernutrition boils down to a basic question: how much stunting can be prevented globally with improved WASH? Various studies have estimated the WASH‐attributable disease burden over the last two decades (Clasen *et al.* 2014b), with various single or multiple infectious disease outcomes included, such as diarrhoeal diseases, helminth infections, trachoma and schistosomiasis. Of these though, we are aware of only one analysis that has included undernutrition as an outcome in their burden of disease estimate (Prüss‐Üstün *et al.* 2008). This study conducted by WHO categorized the effects of WASH on undernutrition as ‘direct’, meaning attributable deaths resulting from protein energy malnutrition, and ‘indirect’, meaning attributable deaths resulting from increased susceptibility to infectious diseases as a result of undernutrition. Taken together, this study estimated that in 2004, a huge number of child deaths – approximately 860 000 – caused by malnutrition might be prevented with improved WASH.

### How can WASH interventions be mobilized to eliminate stunting?

Evidence is growing that sustained exposure to enteric pathogens in early life mediated by poor WASH conditions may have profound effects on child growth and development (Lin *et al.* 2013). In addition, there are multiple social and economic mechanisms by which poor access to WASH can increase the risk of stunting and other forms of undernutrition. In light of this, there is renewed interest in how WASH interventions might be targeted or modified to best support efforts in the nutrition sector (Humphrey 2009a). This has implications for both the nutrition and WASH sectors: for the former, reform may be needed to foster and enable greater cohesion with other complementary sectors, including WASH, and, for the latter, strategies may require modification to support broader efforts to reduce childhood undernutrition.

In countries where rapid progress has been made in recent years, such as Brazil or Peru, one consistent feature has been strong inter‐sectoralism (Dangour *et al.* 2013a). While such inter‐sectoralism is commonly associated with success, fostering such coordination and integration under the MDG has been challenging (Waage *et al.* 2010). Under the Sustainable Development Goals, both the nutrition and WASH sectors have dedicated goals – to ‘end hunger, achieve food security, and improve nutrition and promote sustainable agriculture’ and to ‘ensure availability and sustainable management of water and sanitation for all’ – but dedicated efforts to realize synergies and remove barriers to integration are needed (Waage *et al.* 2015). One opportunity is the Scaling Up Nutrition (SUN) initiative that actively promotes national‐level coordinated action across sectors to end malnutrition. Active in over 50 high‐burden countries, and supported by global agencies, including donor governments, the United Nations and international civil society organizations, the SUN movement provides a basis for the ‘alignment of actions across sectors and among stakeholders’ (SUN 2015) and an entry point for the WASH sector.

The WASH sector, however, faces its own challenges in delivering effective, equitable and sustainable interventions, supported by well‐conceived and resourced national policies and strategies (Bartram & Cairncross 2010). As highlighted in the sanitation trials discussed in the previous text, many WASH interventions are ineffective in mobilizing community uptake and achieving sustained changes in behaviour (Barnard *et al.* 2013). For example, promoting handwashing with soap and basic on‐site sanitation may in principle represent highly cost‐effective public health interventions (Jamison *et al.* 2006), but many of these interventions fail to catalyse significant or sustainable changes in behaviour (Curtis *et al.* 2011). Conversely, while demand is generally high for improved water supplies, many systems fail or perform poorly due to inadequate provision for the management and maintenance of the infrastructure, thereby preventing use where demand is strong. Reducing stunting will require strong WASH programmes that do not repeat old mistakes of supply‐oriented, over‐engineered solutions (Cairncross 1992) nor forget the most important lesson of all that people are unlikely to wash their hands or use sanitation facilities unless they actually want to do so (Cairncross 2003).

It is not clear that traditional WASH interventions or strategies will per se deliver or at least maximize the potential nutrition benefits. Traditionally, WASH interventions have focused on ensuring access to WASH for the general population to improve health and other development outcomes. Under the MDG water and sanitation target – ‘to halve, by the year 2015, the proportion of people without sustainable access to safe drinking water and basic sanitation’ – ‘improved’ water and sanitation were defined with minimum benchmarks of community water supply and basic household sanitation. While much progress has been made under the MDG target – with 2.6 billion gaining access to safe water and 2.1 billion gaining access to adequate sanitation (WHO & UNICEF 2015) – it is unclear whether a water pump located hundreds of metres from the household or a rudimentary latrine are sufficient to protect young children from the growth faltering that results from chronic exposure to enteric pathogens. And, improved hygiene, which can be highly efficacious in reducing diarrhoeal disease, was not included under the MDG target, perhaps because of the difficulty of measuring progress.

### Priorities for a nutrition‐sensitive WASH sector

While more research will help strengthen future nutrition‐sensitive WASH interventions, clear points emerge from the existing evidence base that can help guide the design of nutrition‐sensitive WASH strategies. In essence, the challenge is ensuring that the right people receive the right interventions at the right time. This means ensuring that populations with a high burden of stunting are targeted before or when growth faltering occurs and with appropriate WASH interventions alongside more traditional nutrition‐specific interventions. Reaching and protecting those at risk may require interventions that go beyond the scope of the traditional package of WASH interventions, such as ‘improved’ water and sanitation as defined under the MDG target, to ensure that young children are protected from exposure to enteric pathogens.

As both diarrhoeal disease morbidity and mortality (Walker *et al.* 2013.), and the process of stunting (Shrimpton *et al.* 2001), are concentrated in the first 2 years of life, and this growth deficit is thereafter not recovered, attention should be given to how WASH might limit exposure during this specific window. The recent Cochrane review, discussed in the previous text, validates this focus, reporting that the effect of WASH interventions on stunting was greatest in children aged 0–24 months, in an individual participant data subgroup analysis (Dangour *et al.* 2013b).

Identifying dominant faecal–oral exposure pathways for young children when they are most vulnerable to the deleterious effects of contaminated environments is the first step in identifying those WASH interventions that are likely to be most efficacious. One recent study used structured observation of mother–child couples in Zimbabwe to assess faecal–oral exposure among young children and highlighted the risks associated with the consumption of soil – geophagia – and animal waste in peri‐domestic areas (Ngure *et al.* 2013). A number of recent studies in Mali (Touré *et al.* 2011; Touré *et al.* 2013) and in Bangladesh (Islam *et al.* 2013) have also highlighted the risk to this age group posed by often highly contaminated weaning or complementary food. There has been growing concern, too, about the safe disposal of children's faeces, which are generally not disposed of safely, as they are often considered to be less pathogenic than those of adults, although the reverse may be true (Brown 2003).

WASH interventions that target critical exposure points for young children should be prioritized alongside relevant nutrition‐specific priorities, such as improving infant and young child feeding (WHO & UNICEF 2003). Such WASH interventions might logically include infant food hygiene – the safe preparation, storage and reheating of infant foods – controlling or supervising exploratory play to limit exposure to contaminated soil, fomites and objects (Prendergast & Humphrey 2014) and ensuring that child faeces are disposed of safely. From the perspective of the nutrition sector, this focus and package of interventions is hardly a new concept. Building on a series of seminal studies in the 1970s that demonstrated the effect of repeated infections on growth in early childhood, Mata highlighted the importance of the ‘matro environment’ and the ‘maternal technology’, which included ‘hand‐washing… avoidance of faeces during meal preparation and eating times, (and) adequate preservation of food)’ (1979).

Mirroring a wider debate in the field of international development and global health, there has been an increased focus on equity and non‐discrimination within the WASH sector. Disaggregating MDG progress data by wealth quintile reveals markedly different rates of progress between groups categorized by wealth, with the slowest progress among the poorest (UNICEF 2010). If WASH sector investments are to support efforts to reduce stunting, identifying where stunting is spatially and socially clustered and targeting these populations will be important. As poverty, undernutrition and poor infrastructure often coincide, the potential for positive synergies is high. The public health benefits of targeting WASH interventions at stunted populations are twofold: firstly, that reductions in stunting might be accelerated if WASH interventions deliberately target children at risk, and, secondly, that the impact of WASH on diarrhoea and other diseases might be enhanced by targeting undernourished children who are more susceptible to infection and related mortality (Caulfield *et al.* 2004).

**Key messages**

- Water, sanitation and hygiene (WASH) remain critical interventions for improving maternal and child health.
- A growing body of evidence suggests that WASH are important determinants of childhood stunting.
- WASH influence stunting through direct biological mechanisms by reducing the risk of symptomatic and asymptomatic enteric infections and by social and economic mechanisms, such as diverting household income from food budgets.
- Although more research will strengthen future interventions and policy, there is sufficient evidence to justify the inclusion of WASH within national and international strategies to reduce stunting.
- As the process of stunting and the burden of enteric infections are concentrated in early childhood, WASH policy and programmes should explicitly address this population group in the design and targeting of interventions.

## Conclusions

Improved access to safe and sustainable WASH brings a broad range of well‐documented and widely recognized health and non‐health benefits. In addition, current evidence suggests that WASH can also bring significant gains in tackling childhood undernutrition. Whether it is by the generally better investigated pathways of enteric pathogen exposure or the plausible but less well‐investigated social and economic pathways, poor WASH access is intimately linked to childhood growth and development. Realizing the potential contribution of WASH to global efforts to end stunting will require stronger coordination but may also require that WASH programmes and interventions are modified. While WASH alone will not eliminate stunting, it does have the potential to accelerate progress on eliminating stunting as a critical component of comprehensive strategies.

## Source of funding

The time of both authors was supported in part by the SHARE Research Consortium funded by the UK Department of International Development.

## Contributions

This article was jointly conceived and written by both authors.

## Conflicts of interest

The authors declare that they have no conflicts of interest.
